# Legacy and impact of the 1925 Geneva Protocol: one hundred years of treaties and debates on chemical and biological weapons

**DOI:** 10.3389/fmicb.2025.1685967

**Published:** 2025-12-17

**Authors:** Walter E. Grunden, Olli H. Tuovinen

**Affiliations:** 1 Department of History, Bowling Green State University, Bowling Green, OH, United States; 2 Department of Microbiology, The Ohio State University, Columbus, OH, United States

**Keywords:** biological weapons, chemical weapons, Geneva Protocol, infectious diseases, toxins

## Abstract

This essay examines the legacy and impact of the 1925 Geneva Protocol, which prohibited the use of chemical and biological weapons. This multinational treaty was an important milestone in the history of the non-proliferation of weapons of mass destruction concluded in the wake of the horror engendered by the use of poison gases in World War I. However, the 1925 protocol did not address the issues of verification, manufacturing, stockpiling, and transferring products of chemical and biological weapons or production technologies. A second treaty, the result of the Biological Weapons Convention of 1972, was concluded to address these issues. Despite the apparent success of prohibiting large-scale and widespread use of chemical and biological weapons, violations have occurred, nonetheless, and the treaties remain problematic in numerous ways. The centennial of this historic 1925 agreement and its successor treaties presents an opportunity for reflection upon their efficacy. Given that there have been “limited” episodes of chemical and biological warfare since that time, how can these events be explained in light of the protocol’s prohibitions? This essay argues that the 1925 Geneva Protocol has a mixed legacy and, at best, can be deemed only a qualified success. Other factors, such as political deterrence, scientific and technological limitations, and the problematic nature of these types of weapons may account for the absence of their mass use. The essay aims to serve as an introduction to the historiographical literature with an emphasis on biological weapons and the Geneva Protocol and includes tables as reference guides concerning the types of weaponized pathogens and toxins in question.

## Introduction

The year 2025 marks the centennial anniversary of the 1925 Geneva Protocol (i.e., “Protocol for the Prohibition of the Use in War of Asphyxiating, Poisonous or Other Gases, and of Bacteriological Methods of Warfare”), which prohibited the use of chemical and biological weapons. This multinational treaty was an important milestone in international relations signed in the wake of World War I and the founding of the League of Nations. Some nations, however, found terms of the protocol to be politically problematic, which caused more than a few of them to balk at ratification, as was also the case with the Biological Weapons Convention in 1972, which came into force in 1975 as the “Convention on the Prohibition of the Development, Production and Stockpiling of Bacteriological [Biological] and Toxin Weapons and on Their Destruction.” Since the inception of these agreements, there have been numerous allegations of violations and incidents of deception that have engendered no small amount of controversy. Despite the apparent success of these agreements in prohibiting large-scale and widespread chemical and biological warfare, violations have occurred, nonetheless. The treaties remain problematic in numerous ways. For example, there is still no single international biological warfare treaty that requires mandatory verification of compliance, and signatories have not always complied with voluntary verification, including restricting access to certain toxins and microorganisms that are potential biological weapons. Moreover, the treaties have no real means of enforcement relying, instead, largely upon multilateral trust and self-imposed compliance.

The centennial of this historic 1925 agreement presents an opportunity for reflection upon its efficacy. To what extent can it be argued that the 1925 Geneva Protocol has been successful in preventing the outbreak of chemical and biological warfare over the last century? Given that there have been “limited” episodes of chemical and biological warfare since 1925, how can we explain these events in light of the protocol’s prohibitions? Are there other factors beyond the protocols that may explain why there have not been larger outbreaks of chemical and biological warfare otherwise? This essay argues that the 1925 Geneva Protocol has a mixed legacy and at best can be deemed only a qualified success. Although the signatories have largely abided by the spirit of the agreement, with notable exceptions, other factors may explain why the world has not seen the mass use of chemical and biological weapons beyond merely the 1925 agreement, including deterrence, scientific and technological limitations, and the problematic nature of these types of weapons. This essay will focus largely on the problem of *biological warfare* prohibition. The essay aims to serve as an introduction to the historiographical literature on biological warfare and the Geneva Protocol with additional tables that may serve as helpful reference guides for those less familiar with the subject.

## Defining biological weapons

Following Spencer and Wilcox (1993), we define biological warfare agents as “living organisms, whatever their nature, or infective material derived from them, which are intended to cause disease or death in man, animals or plants, and which depend for their effects on their ability to multiply in the person, animal or plant attacked.” Biological weapons utilizing such agents have several potential uses that can generally be categorized as either *tactical* or *strategic*. Tactical use typically implies deployment for a limited military objective, such as contaminating an enemy’s water supply, weakening enemy forces by spreading illness and disease locally, or using biological agents or toxins to assassinate designated personnel. Strategic use tends to imply larger-scale attacks such as against whole populations or large concentrations of enemy forces. This may include the deliberate spread of infectious diseases that kill indiscriminately and strike terror into the enemy. Attacking crops and livestock to decimate an enemy’s food supply may also be considered a strategic use if deployed on a large scale. Developing biological weapons as a deterrent against enemy use “in kind” may also be considered a strategic use (Cookson and Nottingham, 1969). The United Nations Office for Disarmament Affairs (UNODA) explicitly states that biological weapons may also be used for “political assassinations, the infection of livestock or agricultural produce to cause food shortages and economic loss, the creation of environmental catastrophes, and the introduction of widespread illness, fear and mistrust among the public” (disarmament.unoda.org/biological-weapons/about/what-are-biological-weapons/).

The very nature of biological weapons has led to their development in secrecy. Some countries have had clandestine biological weapons programs that were formally acknowledged to have existed only after their termination. Other nations have continued to suppress information regarding their biological and chemical weapons programs and are suspected of covert continuation despite international agreements. As a result, the subject lends itself to endless speculation, suspicion, and focused intelligence gathering as accurate information is difficult to substantiate in the absence of corroborating evidence and the lack of cooperation by the respective governments where inspection and verification are concerned.

Biological and chemical weapons can serve as both a threat and a deterrence in national defense and regional conflicts. Compared with other forms of offensive weaponry, though often reviled, biological and chemical weapons have historically been attractive as military options because they generally do not cause physical damage at the target site as do more conventional kinetic weapons. While chemical weapons are typically disseminated in munitions such as bombs, mortars, and missiles, or sprayed by aircraft, making their application more easily observable, biological weapons may be delivered in ways that are initially undetectable, making them ideal for clandestine applications, such as poisoning a food or water supply, which may be accomplished by a lone individual properly equipped. Biological warfare agents are particularly susceptible to fluctuations in temperature and generally do not withstand the heat and shock caused by dispersal in munitions. Therefore, dissemination via kinetic weapons, while not always impossible, is less than optimal. Biological warfare agents may also be aerosolized to contaminate larger targets, but this means of deployment has obvious drawbacks for detection (Croddy et al., 2002).

Some biological toxins may evade detection if they are novel biomolecules for which there are no reference compounds, or if their residues are unstable in the environment or biological tissue. Such weapons allow the continued use of affected surfaces and other contacts after the appropriate incubation period, after the contact time has passed, or after the epidemic has subsided. Depending on the biological agent, a time delay in a large-scale attack of the target area may be on the order of days or even weeks rather than seconds or minutes, as is the case with conventional ammunition or chemical weapons. New synthetic or genetically modified toxins can be targeted very narrowly, as in the poisoning of Russian dissidents (e.g., Sergei Skripal and his daughter Yulia in Salisbury, United Kingdom) with Novichok nerve agents in the past 10 years (Chai et al., 2018), in the premediated uses of ricin (castor bean plant, *Ricinus communis*), for example, in the assassination of the Bulgarian dissident Georgi Markov in 1978 (Nehring, 2021; Salisbury and Dew, 2023), and in the anthrax scare targeting select individuals in the U.S. by mail after the terrorist attacks of 9/11 in 2001 (Guillemin, 2011; Bush and Perez, 2012). Another example, widely reported in the media (accessed October 16, 2025)^1^ is the fatal poisoning of the Chinese billionaire Lin Qi in 2020 by former executive Xu Yao, who first tested tetrodotoxin and methylmercury chloride efficacy in his small makeshift animal laboratory.

Prior immunization can provide effective protection against many biological weapons, and antidotes are now available for many chemical and biological poisons provided that the toxic agent can be identified in time. A notable exception is ionizing radiation, as in the case of the assassination of Alexander Litvinenko, a former KGB and FSB agent, who defected to British intelligence in 2001 and succumbed to ^210^Po-induced acute radiation syndrome in 2006 (Dyer, 2007; McFee and Leikin, 2009).

Biological weapons can be classified into several groups of pathogenic agents, including rickettsiae, free-living pathogens, viruses, and biological toxins (Table 1). At least four species of rickettsiae (obligately intracellular bacteria in mammalian cells) have been tested for use in biological weapons. Of the pathogenic bacteria, the most focus has been on thirteen species, especially *Bacillus anthracis*. Over twenty human viruses are potential candidates for mass use as biological weapons. Biological toxins also have potential as agents for warfare because bacterial genes can be manipulated to encode novel toxin properties. Nucleotide sequences of toxin-encoding genes associated with pathogenicity factors are in the public domain and thus readily available. Genetically engineered microorganisms have attracted attention in the biological weapons arena because their toxins can potentially be altered, or the microorganisms may be specifically developed for enhanced toxin production in mass fermentation. Bioengineering and molecular and synthetic biology systems can conceivably be used to design new biological agents that can bypass natural immunity in humans and animals, and artificial intelligence can add powerful extensions in this direction. Genetically modified toxins especially can pose major analytical challenges if they are undetectable by current methods.

**TABLE 1:** Examples of bacteria, viruses and biological toxins that have been considered for potential use in biological warfare or bioterrorism.

Group	Names and designations
Rickettsiae (obligately intracellular bacteria in mammalian cells)	*Coxiella burnetii* (Q-fever); *Rickettsia quintana; Rickettsia prowazekii* (typhus fever); *Rickettsia rickettsii* (Rocky Mountain spotted fever)
Free-living pathogens	*Bacillus anthracis* (anthrax); *Brucella abortus* (brucellosis); B*rucella melitensis* (brucellosis); *Brucella suis* (brucellosis); *Chlamydia psittaci* (psittacosis); *Clostridium botulinum* (botulism), *Francisella tularensis* (tularemia); *Burkholderia mallei* (glanders); *Burkholderia pseudomallei* (melioidosis); *Serratia marcescens* (white pox)
Viruses	Chikungunya virus; Crimean-Congo hemorrhagic fever virus; Dengue virus; Eastern equine encephalitis virus; Ebola virus; Hantaan virus; Japanese encephalitis virus; Junin virus; Lassa virus; Lymphocytic choriomeningitis virus; Machupo virus; Marburg virus; Monkeypox virus; Rift Valley fever virus; Tick-borne encephalitis virus; Variola virus (smallpox); Venezuelan equine encephalitis virus; Western equine encephalitis virus; Yellow fever virus
Biological toxins	Botulinum toxins (*Clostridium botulinum*); Enterotoxins (C*lostridium perfringens, Staphylococcus aureus)*; Conotoxin (marine snails); Ricin (castor beans); Saxitoxin (dinoflagellates); Shiga toxin (enterohemorrhagic *Escherichia coli*); Tetrodotoxin (globe fish, puffer, and others in the family of Tetraodontidae); Verotoxin (enterohemorrhagic *E. coli*); Microcystin and other cyanobacterial toxins

Information has been sourced from the Australia Group and U.S. Federal Government agencies. (www.dfat.gov.au/publications/minisite/theaustraliagroupnet/site/en/objectives.html).

Many microorganisms have been considered for biological warfare because of their virulence and pathogenicity (Wheelis et al., 2006). Some have natural biological vectors for dissemination such as insects, murine rodents, birds, and swines. Zoonotic diseases combined with resistance to antimicrobial drugs are of major concern. Animal and plant pathogens have received international attention because the disruption of animal husbandry or crop production would have strategic regional and national significance. National and international culture collections and databases serve as public repositories of almost all microbial cultures, nucleotide sequences of target genes, 16S rRNA genes, and genomic data published in peer-reviewed scientific and medical literature. Examples of databases are, among others, the CNGBdb, ENA, and NCBI (The China National GeneBank DataBase, European Nucleotide Archive, and National Center for Biotechnology Information, respectively). These culture collections were once available to the public at large without much scrutiny and precaution, but accessibility to virulent pathogens, such as *Bacillus anthracis*, is now severely restricted. To date, some countries remain in embargo or are required to participate in an individual validated licensing system to receive dual-use equipment or technical data that can be potentially used for the design, development, production, or use of biological weapons. Although there are many analytical methods (Table 2) that have been introduced for field and laboratory detection and investigation, the lack of access to suspected facilities has hampered inspections, not to mention incidents of deliberate concealing of evidence of biological weapons (Russell and Vogler, 2000).

**TABLE 2:** Examples of categories of detection methods for biological warfare agents.

Category	Comments
Microscopy, fluorescence microscopy	Insufficient for full identification; low sensitivity; unsuitable for food, soil, and sediment samples, lack of specificity, can be combined with FISH technique (fluorescent *in situ* hybridization) to high specificity
Cultural recovery	Time-consuming, cultural recovery unknown. Requires containment facilities: in E.U. and U.S. biosafety levels (BSL) 1 through 4, in Canada containment levels 1 through 4.
Immunoassays	Numerous immunoassay techniques available; non-specific binding and false negatives and positives require confirmatory methods
Nucleic acid-based methods	In principle, any nucleotide sequence can be detected; PCR requires only a relatively small amount of target sequence (100 copies or even less). Sequencing is essential.
Physical and chemical methods	IR spectroscopy and mass spectrometry for signature molecules and peptide fingerprinting

Before diagnosis, representative samples must be retrieved, documented, and transferred to an analytical facility. Samplers and sample quantities depend on the matrix; for example, air, soil, sediment, freshwater, potable water, food, biological tissue. Sample preservation may be essential, and laboratory preparedness and analytical manuals are required. Current literature is replete with analytical and diagnostic methods for biological targets in each category.

The Centers for Disease Control and Prevention (CDC) has established three categories of pathogenic microorganisms ranked in order of their potential for weaponization and bioterrorism, as well as their general risk to public health and national security (Table 3). Sampling and rapid diagnosis are among key initial actions in this kind of public health threat and incident. The CDC is a partner in the Laboratory Response Network, which provides rapid access to federal, state, and local public health, military, food testing, environmental, and veterinary laboratories, their analytical expertise and equipment, and data exchange. The Network also has extensive international partners (e.g., The International Pathogen Surveillance Network), that can provide analytical and intelligence support in testing and identification protocols and data sharing. Similarly, Great Britain and the E.U. countries have developed up-to-date diagnostic capabilities and coordination in information sharing and are network-connected in bio-surveillance for biological agents and biothreats. Intelligence agencies have major roles in the reconnaissance for threats of biological and chemical agents.

**TABLE 3:** CDC prioritization of diseases and pathogenic agents that pose potential bioterrorism: threats (https://www.cdc.gov/niosh/ershdb/agentlistcategory.html, accessed October 1, 2025).

Category and definitions	Disease/pathogenic agent	Pathogen
**A**		
Easily disseminated or transmitted from person to person. High mortality rates and the potential for major public health impact. May cause public panic and social disruption. Requires special action for public health preparedness.	Anthrax	*Bacillus anthracis*
	Botulism	*Clostridium botulinum* toxin
	Plague	*Yersinia pestis*
	Smallpox	Variola major
	Tularemia	*Francisella tularensis*
	Hemorrhagic fever	Filoviruses (Ebola, Marburg); Arenavirus (Lassa, Machupo)
**B**		
Moderately easy to disseminate. Moderate morbidity rates and low mortality rates. Require specific enhancements of CDC’s diagnostic capacity and enhanced disease surveillance.	Brucellosis	*Brucella* spp.
	Epsilon toxin	*Clostridium perfringens*
	Food safety threats	*Salmonella* spp., *Escherichia coli* O157:H7, *Shigella* spp.
	Glanders	*Burkholderia mallei*
	Melioidosis	*Burkholderia pseudomallei*
	Psittacosis	*Chlamydia psittaci*
	Q fever	*Coxiella burnetii*
	Ricin toxin	*Ricinus communis* (castor beans)
	Staphylococcal enterotoxin B	*Staphylococcus aureus*
	Typhus fever	*Rickettsia prowazekii*
	Viral encephalitis	Alphaviruses, such as eastern equine encephalitis, Venezuelan equine encephalitis, western equine encephalitis
	Potable water safety threats	*Vibrio cholerae*, *Cryptosporidium parvum, Giardia parvum*
**C**		
Regulated availability. Ease of production and dissemination. Potential for outbreak with high morbidity, mortality rates, and major health impact.	Emerging infectious diseases; some are zoonotic diseases	Examples: Nipah virus, Hantavirus, Lyme disease, Zika virus, HIV, Avian influenza virus, West Nile virus, Covid-19, MERS, SARS

Smallpox ranks highly on any list of potential biological warfare agents. Although this virus has been eradicated from the world population, two stocks of smallpox are known to exist in high-level isolation facilities: one at the CDC, Atlanta, GA, and the other at the Vector Research Unit, Koltsovo, Novosibirsk Oblast, Russian Federation. Both locations possess over one hundred strains of this variola virus, genus *Orthopoxvirus*. There remains great concern about the possibility of the virus finding its way accidentally or deliberately into the hands of terrorists who would not hesitate to use it for nefarious purposes. Genetically very closely related to the smallpox virus is the camelpox virus, which is endemic among camels, but does not appear to pose a health hazard to humans such as camel handlers who are in frequent contact with the natural host. It is not known whether camelpox virus poses a risk to those humans who have had no previous contact with the natural host. According to documents prepared by UNSCOM (United Nations Special Commission) inspectors in 1995, Iraq attempted to weaponize camelpox at the height of its biological weapons program. Other genetically related members of this genus that can infect humans include cowpox, vaccinia, monkeypox, and rabbitpox viruses (Tucker, 2001). These viruses tend to have distinct geographical patterns of distribution and can be virulent in humans, but to date, none are known to have been released deliberately to infect humans or animals.

The World Health Organization (WHO) initially pushed for a 1999 target date for destruction of the Russian and American smallpox cultures, but this date was postponed indefinitely due to international debate over whether the stocks should be destroyed or remain in storage indefinitely. Routine vaccination against smallpox ceased in the U.S. and worldwide in 1978–1980, but in the year 2000, the CDC awarded a contract to a biotechnology company to produce a smallpox vaccine, reflecting a growing concern for security in the event of an act of bioterrorism. A French company, Aventis Pasteur, turned over to the U.S. government some 85 million doses of a similar vaccine that it had discovered in its freezers, where the stock had been stored frozen for about 40 years without a loss of biological activity. Smallpox vaccine production in the U.S. was recommenced in 2003 to provide protection for military personnel, health care workers, and emergency response teams. Smallpox vaccine MVA-BN can be used to protect against vaccinia virus and monkeypox virus, which caused a global epidemic in 2022–2023 (Pischel et al., 2024).

Among other animal diseases that could potentially be used in biological warfare, foot-and-mouth disease is particularly infectious and transmissible in hoofed animals, including cattle, pigs, sheep, and goats. The disease is caused by a virus of the apthovirus group, which has multiple serotypes with no cross-immunity. The virus is contagious and transmitted by air, contaminated clothing, boots, and equipment. It can remain viable in manure or straw for extended periods. Foot-and-mouth is considered a global disease as occurrences have been reported in multiple countries (Humphreys et al., 2025). A naturally occurring outbreak of foot-and-mouth disease in the U.K. in 2001 spread within weeks to many regions of the country. As a result, over 2,000 cases of the disease were confirmed and about four million animals were slaughtered, causing economic losses that were estimated at £8 billion (about £15 billion adjusted to July 2025). Its long-term persistence in the U.K. remains an issue of concern (Knight-Jones and Rushton, 2013). A similar outbreak in Taiwan in 1997, with the virus possibly introduced through smuggled meat or animals, devastated the pig farming industry and resulted in economic losses of billions of dollars. Fortunately, there were no human casualties of the disease involved in either outbreak, which was consistent with the lack of virulence of the virus in humans. In January 2025, foot-and-mouth disease was detected in a water buffalo farm in Brandenburg, Germany, but it was contained successfully with strict control measures and a monitoring program. In March 2025, the disease was detected in cattle in Hungary and later in the neighboring Slovak Republic (accessed 18 October 2025).^2^

Several plant pathogenic candidates also have been used for biological warfare. These have included potato blight (caused by *Phytophthora infestans*) and bacterial soft rot (*Erwinia caratovara*) affecting cabbage, carrots, onions, and potatoes. Stem rust of wheat (*Puccinia graminis* f. sp. *tritici*) has proven a good candidate because *Puccinia* spores can be released in the air over a large area. Other plant pathogenic fungi, bacteria, and viruses known to cause diseases in staple crops include common bunt (*Tilletia* fungi), covered smut (*Ustilago hordei*), black stem rust (*Puccinia graminis*), bacterial blight (*Pseudomonas savastanoi*), and white mold (usually *Sclerotinia sclerotiorum*), all of which have potential as biological weapons to cause crop loss as wild types or genetically modified pathogens with increased virulence. Plant pathogenic viruses have been ranked in order of their economic and scientific importance with the top five being the tobacco mosaic virus, tomato spotted wilt virus, tomato yellow leaf curl virus, cucumber mosaic virus, and potato virus (Scholthof et al., 2011).

Colorado potato beetles (*Leptinotarsa decemlineata*) were under consideration for weaponization in WWII. In 1939, France developed a program for their mass production, but this was cut short when Germany invaded the country (Lockwood, 2009). Subsequently Germany developed capacity for the mass production and dispensation of the beetle in WWII. After trials of dispensing them aerially, Germany dispersed them in Isle ofWight in 1943 and later in Sussex, but evidence for the latter is in doubt. The outcome of these beetle attacks remained local, but it incentivized the U.K. and U.S. to develop capacity for mass production of the Colorado potato beetle. There is no physical evidence that this entomological weapon was used during the Cold War, although the Soviet Union accused the U.S. of targeted dispersal (Lockwood, 2009).

## A summary history of biological warfare through World War II: the formative years

Disease transmission has been a tactical option for military personnel, rebels, insurrectionists, and terrorists for many centuries, with suspected incidents dating back as far as 300 B.C. when the Greeks, and later the Romans and Persians, used the rotting carcasses of animals to contaminate the drinking water sources of their enemies (Robinson, 1971). Diseases were often associated with foul odors emitted from decaying carcasses and likely gave rise to the idea of contaminating enemy environments to gain a military advantage. Fighting forces throughout the world used carcasses as well as corpses of captured soldiers to pollute potable water supplies from the classical age to medieval times and to the modern era (Geissler and van Courtland Moon, 1999). But biological warfare would not become a truly viable component of any modern military arsenal until the development of biology, medicine, and hygiene as modern fields of science (Grunden, 2005).

The history of biological warfare, however, is replete with reports of deliberate attempts to weaponize diseases even well before the germ theory of disease was discovered or well understood, some of them historical and some only anecdotal. Among the more infamous of historically documented cases of “purposeful infection” occurred during the British colonization of North America when Sir Geoffrey Amherst, the British Commander-in-Chief in North America, urged his officers to use the deliberate spread of smallpox as a stratagem to reduce the numbers of indigenous enemies. Colonel Henry Bouquet, then serving as the ranking officer on the Pennsylvania frontier, wrote to Amherst of his intent to “inoculate the Indians with some blankets that may fall in their hands,” an incident that seems later to have become conflated with anecdotes of European (civilian) settlers allegedly spreading smallpox by gifting contaminated blankets, a charge much more difficult to substantiate (Oldstone, 2010). During the American Revolutionary War (1775–1783), British troops were vaccinated (variolated) against smallpox to prevent the spread of the disease among their own soldiers, while at the outset of the war Colonial troops remained vulnerable. Outbreaks among the Colonial troops inevitably occurred, and fearing deliberate infection by the enemy, in 1777, General George Washington ordered the inoculation of the entire Continental Army (Oldstone, 2010). Whether infection spread to Colonial forces by intent or incidental exposure in combat cannot be determined for certain.

During the U.S. Civil War (1861–1865), there were alleged incidents of premeditated pollution of drinking water poisoned with the carcasses of sheep and pigs. There were also allegedly plans by the Confederates to send clothing contaminated with yellow fever to the opposite side. The plan failed because yellow fever virus is mosquito borne and not transmitted through contaminated clothing collected from diseased people. Both sides also incurred considerable losses of war horses because of outbreaks of glanders (Koenig, 2006). The Civil War also marked the first instance of alleged use of an insect as a weapon of war. It was alleged (but never proven beyond doubt) that the Union deliberately introduced the Harlequin bug, *Murgentia histrionica*, to the South to cause crop damage. The insect attacks crucifers of all kinds and many other edible vegetables and can also destroy field crops and fruit trees. The South had experienced devastating outbreaks of communicable diseases and malaria due to unhygienic conditions during the war, or so Northerners believed. The Union also blockaded Confederate ports to curtail access to shipments of medicine, especially quinine, as well as food and clothing, thus exacerbating unsanitary field conditions in the camps (O’Flaherty, 1955).^3^

In 1874, representatives from fifteen European states convened in Brussels to address methods and means of combat and warfare. Two documents were drafted as a result of the Brussels Conference: “The Final Protocol of the Brussels Conference 1874” (a.k.a. Brussels Declaration), and “Project of an International Declaration Concerning the Laws and Customs of War.” Article 13(a) of the latter prohibited “employment of poison or poisoned weapons.” Some of the participant governments of the conference, however, did not ratify the declaration and refused to abide by the convention. The International Institute of Law in Geneva undertook an extensive review of the Declaration, which resulted in the publication of the “Manual of Law and Customs of War,” adopted in Oxford (Oxford Manual) in 1880. Two subsequent conventions at The Hague, in 1899 and 1907, which explicitly prohibited the use of poison gases, formalized the agreements resulting in “Conventions on Land Warfare,” later annexed with “Regulations,” which were largely based on the terms previously agreed upon in the Brussels Declaration and the Oxford Manual. Europe at the time was increasingly experiencing political and national tensions. In retrospect, it appears as though the potential role of microbes in spreading communicable human diseases and causing epidemics was becoming widely recognized among the European powers, and these international conventions reflected these growing concerns. This was the period when the ubiquity of microbes and their role in diseases was researched in European countries especially. Development of new biological methods, culturing, and classification opened new vistas of microbial biology in health and disease as well as in many other areas of human welfare.

Of greater and more immediate concern after the turn of the twentieth century, however, was chemical warfare in the form of poisonous gases. Chlorine gas, for example, was deployed on a large scale by German troops on April 22, 1915, at the Second Battle of Ypres in Belgium during World War I, a clear violation of the Hague agreements. In the German Empire, Fritz Haber, the 1918 German Nobelist (Chemistry), who discovered the method to produce ammonia from nitrogen and hydrogen gases in the Haber-Bosch Process, was instrumental in developing heavy chlorine gas used for offensive purposes (Willstätter, 1965; Braterman, 2012). Other principal belligerents of the war, including France, the U.K., Russia, Austria-Hungary, Italy, and the U.S., subsequently deployed chemical weapons themselves, including tear gas, chlorine, phosgene, sulfur mustard, and hydrogen cyanide gases (Spiers, 1986, 2010; Brown, 2006). Germany also used bacterial weapons in attempts to transmit anthrax (*Bacillus anthracis*) and glanders (*Burkholderia mallei*) to horses and other animals and to contaminate the animal feed of the enemy forces, but these apparently did not yield successful results (Geissler and van Courtland Moon, 1999). Following the capitulation of Germany on November 11, 1918, the principal belligerents met in Versailles to conclude The Treaty of Paris, which was drafted in 1919 and took force in 1920. Article 171 of the Treaty specifically prohibited the use of “asphyxiating, poisonous or other gases and all analogous liquids, materials or devices,” while Article 172 demanded the destruction of all existing stockpiles of chemical weapons. No mention, however, was made of biological or bacteriological weapons (Geissler and van Courtland Moon, 1999).

The Geneva Protocol, signed on June 17, 1925, is a historical milestone because it was the first formal, international agreement concerning the prohibition of both chemical *and* biological weapons. Although the signatories were in general agreement about the horrific nature of these weapons, various aspects of international law as engendered by the Geneva Protocol were not sufficiently clarified, leaving many nations hesitant to ratify. (Significantly, although all parties in attendance became signatories, most nations did not ratify the protocol until years or decades later, including Japan in 1970 and the U.S. in 1975.) According to historian Edward M. Spiers, the protocol “failed to address the R&D, production, possession or transfer of such weapons.” Moreover, it “avoided any reference to how the agreement could be verified or enforced,” essentially leaving it binding “only in relation to other states who were a party to the protocol” and it would “cease to be binding whenever enemy states used gas warfare.” In short, the protocol was rendered little more than a “no first use” agreement (Spiers, 2010). While the Geneva Protocol came into force in 1928 and was registered with the League of Nations in 1929, it remained a problematic document whose enforcement remained unresolved (Dorsey, 2024). It was arguably as toothless as the League of Nations itself.

While most of the principal belligerents of World War I established programs, departments, or agencies to oversee the development and production of chemical weapons, none of them established formal biological weapons programs at that time. As the specter of fascism arose in Europe, however, that would begin to change. By the 1930s, most had returned to producing poison gases in mass quantities, and research and development on innovative systems for delivery and defense were underway in most every developed nation. Italy’s use of mustard gas against Abyssinian forces in 1935 revealed the impotence of both the protocol and the League of Nations to prevent even the first use of chemical weapons in battle (Robinson, 1971; Harris and Paxman, 2002). With another global conflict looming on the horizon, the major powers explored all manners of weapons, even those ostensibly outlawed.

World War II brought about major investments in biological weapons programs among the primary belligerents. It should be remembered that the Geneva Protocol effectively banned only the first use of these weapons, and, as such, it did not act as a deterrent to research and development, or even production. In response to intelligence reports implicating Germany in developing bacteriological weapons, Sir Maurice Hankey, Secretary to the Cabinet and the Imperial Defense Committee, took the lead in promoting a biological weapons research program in the U.K., resulting in the establishment of the Microbiological Warfare Committee in October 1936. The program began on a defensive footing until the outbreak of war in September 1939, whereupon it took on a more offensive posture. In 1940, a modest biological weapons research facility was established at the Porton Down Chemical Defense Experiment Station near Salisbury, Wiltshire, under the direction of Britain’s leading pathologist and microbiologist, Dr. Paul Fildes. A Biology Department was created at Porton Down that year to oversee research (Hammond and Carter, 2002). Anthrax was an early focus of the British biological weapons program. In the event of a German biological weapons attack, the U.K. planned to respond by dropping anthrax-laced cakes made of ground linseed meal across the German heartland. The plan to infect sheep and cattle, designated “Operation Vegetarian,” never materialized. Extensive experiments with anthrax were conducted on Gruinard Island (57.8868° N, 5.4673° W), where sheep were exposed to airborne *B. anthracis* or, in other instances, were shot with hollow bullets filled with anthrax spores. None of the 80 sheep survived. Due to the extent of contamination, Gruinard remained quarantined until 1986, when decontamination efforts were undertaken. Gruinard Island was declared anthrax free in 1990 by the U.K. Ministry of Defence (Manchee et al., 1981; Balmer, 2001; Harris and Paxman, 2002; Spiers, 2010). The U.K. Ministry of Defence had requisitioned the island for £500 in 1940 and sold it back to the heirs of the original owner for the same amount of sterling in 1990 (Aldhous, 1990).

In the U.S., the Chemical Warfare Service (CWS) received critical upgrades and increased financial support with budget allocations rising from $2 million in 1940 to over $1 billion in 1942 (Robinson, 1971). In 1943, a formal biological weapons program was established under the auspices of the CWS. The War Research Service (WRS) was established to serve as an advisory body on biological weapons policy, while R&D remained under the purview of the CWS. In April 1943, the Biological Weapons Research and Development Center was established at Camp Detrick in Frederick, Maryland. Later known as Fort Detrick, this site became the first biological weapons research and development facility in the U.S., employing some 4,000 staff members and researchers with supporting facilities for production and ordnance testing built at the Vigo Ordnance plant near Terre Haute, Indiana, Horn Island in Mississippi Sound and Granite Peak near the Dugway Proving Grounds in Utah (Robinson, 1971; Brophy et al., 1959). The U.S. biological weapons program pursued research on anthrax, botulism, brucellosis, psittacosis, tularemia, and glanders. In addition to these human and animal diseases, the U.S. also pursued research into several plant pathogens, continuing well into the Cold War era.

The Soviet Union (USSR, Union of Soviet Socialist Republics) established a formal biological weapons research program as early as 1925 with the formation of the Military Chemical Agency under the direction of Yakov Moiseevich Fishman. The Ministry of Defense and Ministry of Health, however, remained responsible for the oversight of biological weapons research at no less than thirty-five institutions throughout the country, ranging from the Moscow Institute of Epidemiology and Microbiology to comparable facilities, for example, in Leningrad (today St. Petersburg) and Koltsovo, Novosibirsk Oblast. Testing grounds were erected in isolated areas such as Gorodomlya Island in Lake Seliger for greater secrecy and safety. Soviet scientists investigated plague, anthrax, tularemia, typhoid, glanders, cholera, and foot-and-mouth disease (Robinson, 1971; Geissler and van Courtland Moon, 1999; Harris and Paxman, 2002; Leitenberg et al., 2012). It has been alleged that the Soviet Union deployed tularemia (*Francisella tularensis*) in the battle of Stalingrad (today Volgograd) in WWII (Alibek, 1999). However, this allegation has not been confirmed by independent sources, and tularemia of natural causes was one of the diseases afflicting both Soviet and German troops. The Soviets continued to invest in biological weapons R&D in the postwar era, but the onset of the Cold War made verification of their programs’ activities difficult to verify.

Despite having ratified the Geneva Protocol in 1929, and regardless of limitations imposed upon it concerning rearmament, by the early 1940s, Germany had recovered much of its chemical weapons production capacity and had branched out into biological weapons R&D as well, though on a comparatively much smaller scale. Germany’s biological weapons program, such as it was, remained decentralized and split largely between agencies and institutions under Dr. Heinrich Kliewe as director of research in the Office of the Surgeon General of the Wehrmacht (Chef des Wehrmachtsanitätswesens), and Professor Kurt Blome, director of the Kaiser Wilhelm Institute’s Center for Cancer Research. The German BW program never equaled that of the U.K. or U.S., as it was never made a priority. Postwar U.S. intelligence assessments attributed this to “Hitler’s personal opposition to the use of biological weapons,” though the supposed reasons for that disposition remain a point of debate among historians (Geissler and van Courtland Moon, 1999; Grunden, 2005; Spiers, 2010). Although Hitler was reportedly vehemently opposed to any offensive use of either chemical or biological weapons—ostensibly due to his own experience in having sustained injuries from a poison gas attack while serving as a corporal in WWI—this did not stop the Nazi regime from developing new forms of nerve gases, such as *sarin* and *tabun*, nor did it prevent them from testing their efficacy in experiments upon millions of Jews, Roma, and others deemed “*Untermenschen*” who were murdered in the numerous death camps in Germany and Poland, where most of the chemical and biological weapons experiments were undertaken and where such poisons were used for mass extermination. Rather, it is more likely that Hitler chose not to use these weapons for fear of retaliation in kind or due to logistical complications that made deployment disadvantageous otherwise (Schmaltz, 2017; Schmidt, 2015).

Of all the principal belligerents of WWII, it was Japan that arguably violated the terms of the Geneva Protocol with first-use strikes utilizing both chemical and biological weapons; yet the perpetrators of these acts—with few exceptions—escaped justice. Japan’s foray into biological weapons research began in 1932, with Dr. Shiro Ishii, a microbiologist, then a senior army surgeon, third class (rank of major), who actively lobbied his superior officers to establish a biological weapons program. Ironically, Ishii was inspired by nothing less than the Japanese delegation’s own report on the Geneva Protocol, which, he noted, specifically outlawed chemical and biological weapons. Biological weapons, Ishii argued, must have significant potential, otherwise, why would they have been prohibited? After undertaking a study of other nations’ efforts in this field, Ishii concluded that Japan must pursue a biological weapons program or risk falling behind. The army brass agreed. Under the auspices of the Kwantung Army, a branch of the Imperial Japanese Army stationed in China, Ishii established two small-scale research facilities in the occupied area of China’s northeast provinces (Manchuria), then known as Manchukuo, a puppet state under Japanese control from 1932.

In 1936, Ishii’s operation was upgraded and officially designated the “Kwantung Army Epidemic Prevention and Water Supply Unit,” also known as “Unit 731.” The following year, the Kwantung Army built an extensive research center for Ishii to continue his work. Located on the outskirts of the city of Harbin, the Pingfang complex ultimately grew to about 3,000 staff members, including medical doctors and microbiologists recruited from Japan’s most prestigious institutions of higher education. Although mainly interested in plague, Ishii and his collaborators also conducted research on weaponizing smallpox, botulism, brucellosis, cholera, and dysentery. Together with Unit 100, the “Hippo-Epizootic Detachment,” they also cultivated anthrax and glanders for use against horses, sheep, and cattle. Research was also conducted on developing anti-plant agents, such as weaponizing *Puccinia helianthi*, a fungus also known as “red rust” and “common rust,” that is particularly harmful to numerous types of crops. The fungus is autoecious, that is, its life cycle is all within the same host plant.

In 1939, Ishii’s Unit 731 detachment was mobilized to deploy biological weapons against the Soviet Red Army during the Battle of Khalkin-Gol (Nomonhan) near the border of Mongolia. To slow the advance of the Red Army, Unit 731 contaminated their main source of fresh water—the Khalkin-Gol River—with typhus, paratyphus, and cholera. The results were mixed but sufficiently encouraging for the Imperial Japanese Army to establish several more biological weapons units throughout its empire. For the duration of the war, Japan continued to engage in biological warfare, especially throughout China, but allegedly reaching as far as Burma, Thailand, Indonesia, and the Crown colony of Singapore (now known as the Republic of Singapore), resulting in deaths and casualties—largely civilians—ranging from the tens to the hundreds of thousands. Japan gained a decisive edge over the other principal belligerents in biological warfare because of its unfettered exploitation of human subjects, many of whom were subjected to experimental vaccines and treatments—and even vivisected (i.e., autopsies conducted on live subjects)—to observe the course of the various diseases and their effects on the body. An estimated 3,000 prisoners are believed to have perished at Pingfang at the hands of Unit 731 personnel (Williams and Wallace, 1989; Harris, 1994; Grunden, 2005).

After the war, in one of history’s greatest miscarriages of justice, the U.S. granted immunity from prosecution to Ishii and other members of Unit 731 in exchange for their research data, the full extent and value of which remained unknown to U.S. intelligence and the Joint Chiefs of Staff when the deal was made. Only a dozen or so members of Unit 731—those unfortunate enough to have been captured by the Red Army as it overtook Harbin in the closing days of the war—ever faced a tribunal, a trial conducted by the Soviet Union in the city of Khabarovsk in 1949 (Materials, 1950). None of the perpetrators of biological warfare faced justice at the International Military Trial for the Far East (i.e., Tokyo Trials). Nor were any Japanese leaders brought to justice for conducting chemical warfare, which the Japanese Army resorted to on more than 2,000 occasions in the China Theater alone. Given the close connection between the two types of warfare and the units deploying them, the U.S. Joint Chiefs, in consultation with General Douglas MacArthur, who oversaw the occupation of Japan as the Supreme Commander for the Allied Powers in the Pacific, made the decision not to pursue war crimes charges for the use of chemical weapons lest that investigation reveal the deal made with Ishii. As a result, many of those most responsible for first-use incidents of biological and chemical warfare went unpunished (Guillemin, 2017; Grunden, 2005). The Geneva Protocol had utterly failed to prevent Japan’s use of these weapons in China and throughout Asia.

## The Cold War (1947–1991): the bipolar years

The Geneva Protocol of 1925 did not prevent the Axis powers from resorting to chemical and biological warfare during World War II. Italy and Japan both deployed chemical weapons in battle, and one can make an argument that Nazi Germany violated the spirit of the Geneva Protocol by murdering Jews with poison gases during the Holocaust. Japan is also known to have engaged in biological warfare throughout its empire during the war. Given these facts, it would have been naïve for any of the major powers to assume that the protocol would prove more effective in the postwar era. All the major Allied powers of WW II continued research and development of chemical and biological weapons, which was not prohibited by the Geneva Protocol (Wheelis et al., 2006). In the U.K., research in biological weapons continued at Porton Down, where, in 1945, the Biology Department was renamed the Microbiological Research Department (MRD). The focus at the MRD was twofold including “vigorous laboratory investigation of potential agents” and large-scale field tests “with pathogens, toxins, or their simulants,” though ostensibly more care was taken to reduce risk by working with non-pathogenic bacteria where possible, such as *Serratia marcescens* and *Bacillus atrophaeus* (formerly *B. globigii*), which were used as simulants for pathogens (Guillemin, 2005; Hammond and Carter, 2002). Pilot plants were erected for their mass production. Experiments with actual pathogens, however, were generally preferred or even necessary for evaluating “real life” conditions. In 1946, the MRD was redesignated the Microbiological Research Establishment (MRE), which continued to operate under the auspices of the Ministry of Defence.

In coordination with the Royal Navy, the British Army, and U.S. military forces, Porton Down conducted open-air sea trials using a variety of live pathogens from 1948 through the early 1950s. “Operation Harness” entailed open-air sea trials off the coasts of Antigua and St. Kitts in the Caribbean ostensibly to avoid large-scale contamination such as what occurred on Gruinard Island (Hammond and Carter, 2002). Among the major discoveries in these trials were the short half-lives of biological agents disseminated in aerosols in the open environment. In some cases, sunlight was found to reduce the half-lives from minutes to seconds. The U.K. discontinued open-air testing in the mid-1950s and, in 1959, declared it would terminate its offensive biological weapons research altogether, though bacteriological experiments using *B. atrophaeus*, *Escherichia coli 162*, and *S. marcescens* as models to simulate the survival and dispersion of potential warfare agents were carried out throug the 1960s. All three bacteria were reputed to be harmless and non-pathogenic. Some experiments simulated attacks on urban areas, including the release of simulants at ground level in the Central London area and elsewhere in southern England. As part of these trials, simulant bacteria were introduced to ventilation systems in public buildings and even into the London Underground system. According to the U.K. Ministry of Defence, the releases did not constitute a public health hazard because the virulence of the test bacteria was greatly lowered (Balmer, 2001). In hindsight, however, the potential of infection could not have been unambiguously ruled out. Some facilities in Porton Down were also used in early trials of antibiotic production (licheniformin from *Bacillus licheniformis*) in collaboration with British pharmaceutical companies in the civilian sector. In 1979, the Centre for Applied Microbiology and Research (CAMR) was opened in Porton Down and the MRE was closed. MRE facilities were transferred to CAMR, which operated under the auspices of the U.K. Public Health Laboratory Services and now focused largely on vaccine development. A small Defence Microbiology Division was created within CAMR and has remained throughout the years as a safeguard and advisory resource (Carter, 1992). CAMR maintains the National Collection of Pathogenic Viruses and is part of the Defence Science and Technology Laboratory on the Porton Down campus, which is aligned with the Ministry of Defence and responsible for ensuring strategic defense and security in the U.K. (DSTL, 2025). The Porton Down Science Park on the campus houses several biotechnology companies in the defense, security, and health sectors.

In the U.S., while much of the focus turned to the nuclear arms race with the Soviet Union, advocates in the military sought to elevate biological weapons research “to approximate nuclear scale,” and research and development in chemical and biological weapons continued apace (Guillemin, 2005). The U.S. Chemical Warfare Service, which oversaw biological weapons research through WW II, saw its budget significantly reduced with the end of the war but returned to wartime levels in 1947 with the start of the Cold War. Research in biological weapons continued largely in secrecy until allegations of U.S. use of biological weapons surfaced during the Korean War in early 1952. A fact-finding mission to Korea led by the eminent China scholar, Professor Joseph Needham, under the auspices of the World Peace Council, concluded that the U.S. had engaged in biological warfare, a charge the U.S. vehemently denied, and which was never fully substantiated. According to the most recent scholarship on the controversy, made possible by the brief opening of Russian archives shortly after the fall of the Soviet Union, the allegations were fabricated by the KGB (Komitet Gosudarstvennoy Bezopasnosti or Committee for State Security) in collaboration with North Korean and Chinese operatives (Leitenberg, 1998, 2008). The controversy persists with both positive and negative arguments being advanced in the historiography of the Korean War, with both sides arguing that conclusive, unambiguous evidence has not been presented to disprove their findings. Documented outbreaks of various diseases during the war, including viral hemorrhagic fever, smallpox, cholera, plague, and meningitis among Korean and Chinese soldiers would seem to implicate the United States. Though the U.S. government has acknowledged engaging in biological weapons field tests around this time, it continues to deny that it deployed pathogenic agents or vectors in the Korean War.

The U.S. biological weapons program experienced significant growth in the early 1950s. The Plum Island Animal Disease Center (NY) was established in 1954, and Camp Detrick was renamed Fort Detrick (MD) in 1956. Extensive field testing was undertaken largely using surrogate biological agents to model deadly pathogens. There were many instances of aerial spraying of organisms and simulants over populated areas in the 1950s and into the 1960s (Table 4). In 1950, *Serratia marcescens* was released off the coast of San Francisco at rates up to 5,000 cells/min. In the “St. Jo Program” conducted in early 1953, the U.S. Air Force simulated anthrax attacks on urban areas that included non-infectious aerosol releases over cities such as Minneapolis, MN, St. Louis, MO, and Winnipeg, MB, Canada (Cole, 1988, 1997; Guillemin, 2005). Field tests in the U.S. also included trials with mass breeding and release of potential vectors such as fleas and mosquitoes that could be engaged in transmitting bacterial or viral pathogens (Table 4). In other field experiments, smoke screens were used to disguise aerial tests, and citizens were deliberately misinformed that these exercises involved only harmless smoke that was being tested for use in protecting cities from radar-guided missiles. In New York City, experiments with *Bacillus subtilis* spores showed that release in one underground station could infect the entire underground tunnel due to convection currents and winds (Cole, 1988).

**TABLE 4:** Examples of field test experiments with biological warfare agents and simulants in 1950–1969 in the United States.

U.S. code name	Brief description
Bellwether One	Tally of mosquito bites following release of *Aedes aegyptii*
Bellwether Two	Biting frequency of mosquitoes and spreading on land area
Bellwether Three	Information remains classified
Bellwether Four	Biting activity of two mosquito strains
Magic Sword	Continued Bellwether trials on mosquito dispersal and aerial transport
Big Itch	Aerial dispersal of fleas (*Xenopsylla cheopis*) in munitions
Big Buzz	Breeding, weaponization, and discharge of *Aedes aegyptii* in munitions
Drop-Kick	Uninfected mosquitoes released in residential areas to study their spread and biting behavior.
Grip Iron	Mosquito releases, details not available
May Day	Entomological tests designed to study the dispersal of yellow fever mosquitoes (*Aedes aegypti*) in an urban area. Ground levels tests with mosquito releases
LAC (Large Area Coverage)	Tests to study the dispersal patterns and the geographic range of chemical and biological weapons. The tests were carried out with microscopic ZnCdS particles as the surrogate in several locations
Dark Winter	Simulated initial localized smallpox attack in three municipal locations. The simulation was designed to lead to an inherently unwinnable scenario, whereby the National Security Council could not keep pace with the rate of disease spread and nor determine the origin of the attack and containment
Polka Dot (also known as Trouble Maker)	Field tests with cluster bombs filled with *Bacillus globigii* (now reclassified as *B*. *atrophaeus*)
Sea Spray	*Serratia marcescens* and *Bacillus globigii* (now reclassifieds as *B*. *atrophaeus*) were sprayed over the San Francisco Bay Area to determine how vulnerable a city like San Francisco may be to a bioweapon attack
Dew	Dispersion of aerosol-released biological agents using fluorescent particles and clubmoss (*Lycopodium*) spores
Bacchus	Assessment whether would-be terrorists could covertly build an anthrax production facility and remain undetected. During the 2-year simulation, the facility was constructed and successfully produced an anthrax-like bacterium. The participating scientists were able to make about 1 kg of highly refined bacterial particles covertly
Project 112	Large scale tests in extracontinental sites (Central and South Pacific, and Alaska) with aerosols to disseminate biological and chemical agents that could produce controlled temporary incapacitation. Conjunctively with British, Canadian, and Australian naval forces and intelligence
Whitecoat	Dose-response trials of infectious diseases with U.S. Army volunteers for vaccine development and investigational drugs

Information pooled mostly from Hay (2007), Lockwood (2009, 2012). *Aedes aegyptii* mosquitoes are known vectors of the yellow fever virus and dengue virus. British field tests within the same time frame are summarized by Carter and Balmer (1999), Balmer (2001), Hammond and Carter (2002), and Harris and Paxman (2002).

Other field release experiments, such as “Shady Grove,” which used animals as targets, were performed on barges around the Johnston Atoll site in the South Pacific in the 1960s. Around this time, the vulnerability of U.S. warships to chemical and biological weapons was tested under the aegis of “Project 112” in jet-released aerosol experiments in the Atlantic and Pacific Oceans using chemical markers and surrogate bacteria for anthrax (Regis, 2023). These sea, land and air trials included crew shelters with positive pressure ventilation and immunizations against *Francisella tularensis* and *Coxiella burnetii* in live pathogen experiments (van Courtland Moon, 2009). Non-biological simulants ZnCdS, soap bubbles, and SO_2_ were also used in aerial dispersion studies. These sea trials led to accusations of spreading Newcastle poultry disease in Cuba 1962 and introducing the tobacco blue mold disease (*Peronospora hyoscyami* f. sp. *tabacina*), which devastated Cuba’s tobacco crop in 1979 and 1980 (Lucas, 1980), and releasing the insect pest melon thrips (*Thrips palmi)* in 1997 (Butler, 1997). Melon thrips are widespread and can infest a wide range of edible vegetables and fruit trees in the field as well as in greenhouses (Vázquez and Rodriguez, 1999). Cuba experienced an influx of other agricultural pests and hemorrhagic diseases in the latter half of the century. However, scientific evidence was not presented to support these charges, and the allegations are largely believed to have been politically motivated (Zilinskas, 1999, 2000; Whitby, 2002; Lockwood, 2009).

Significant changes in U.S. biological weapons policy occurred in the 1950s and 1960s, culminating in President Richard M. Nixon’s decision to renounce offensive biological weapons including R&D in the United States in 1969 (U.S. Department of the Army, 1977). He announced that BW research was to be confined to defensive measures only. In the early Cold War era, U.S. biological weapons policy remained on a no-first strike and retaliation basis only footing, a position that aligned with the spirit of the Geneva Protocol, even though the U.S. still had not ratified the agreement. In 1956, in response to what it perceived as bellicose rhetoric emanating from the Soviet Union, the U.S. shifted its position to emphasize that biological weapons could be a viable option in a general conflict. The decision to resort to biological weapons, however, would not be left to commanders in the field but was now explicitly restricted to the president. In 1961, institutional changes initiated by Secretary of Defense Robert McNamara saw biological weapons R&D being subsumed by Munitions Command under the purview of the Army Material Command, giving the U.S. Army exclusive rights to biological weapons development. Field testing was expanded and accelerated at this time, including the aforementioned operations, as well as “Magic Sword,” which tested the spread of dengue and yellow fever in the Pacific using simulants, “Yellow Leaf,” which tested the viability of various biological agents in jungle terrain, and trials of chemical agents VX and sarin conducted under the umbrella of Project 112 from 1962 to 1970 (Guillemin, 2005; Hersh, 1968).

Facing increasing pressure at home and abroad over the use of riot-control agents (CS tear gas) and defoliants such as Agent Orange (containing carcinogenic 2,3,7,8-tetrachlorodibenzo-*p*-dioxin as an impurity) in the Vietnam War, in November 1969, President Nixon declared that the U.S. would no longer pursue biological weapons R&D for offensive purposes and would restrict all work to defensive purposes only. Existing stockpiles would be destroyed, and further restrictions would be placed on CW production as well. Nixon also declared his intent to ratify the Geneva Protocol. Although this was clearly progress, there were limitations. While Nixon ordered the discontinuation of the use of defoliants in Vietnam in 1970, he sustained the army’s request to allow the continued use of CS with restrictions. On April 10, 1972, Nixon signed the multilateral Biological Weapons Convention (BWC), which with a later amendment to include toxins came to be known as the “Convention on the Prohibition of the Development, Production and Stockpiling of Bacteriological (Biological) and Toxin Weapons and on their Destruction,” or more commonly, the Biological and Toxin Weapons Convention (BTWC), which became effective on March 26, 1975. But by the time the BTWC came into effect, and the U.S. Senate ratified the Geneva Protocol on April 10, 1975, Nixon had been forced out of office due to the Watergate scandal. Nixon resigned in August 1974 leaving Gerald Ford to officially sign the accord (Guillemin, 2005; Spiers, 2010). According to biological warfare historian Jeanne Guillemin, after 1975, “the dominant issue for those concerned about biological weapons was how and if legal restraints actually prevented secret proliferation” (Guillemin, 2005, p. 131). Evidence suggests that they did not.

Even as the BTWC effectively banned the use of biological agents, the Soviet Union committed itself to an accelerated and expanded offensive biological weapons program. Spurred on by biochemist Yuri Anatolevich Ovchinnikov, on April 24, 1974, Premier Leonid Ilyich Brezhnev issued Order No. 131 DSP, which established the “All-Union Science Production Association,” more commonly known as “Biopreparat.” The activities coordinated under the Biopreparat Program included 47 research and production facilities throughout the Soviet Union. At its height, Biopreparat employed over 30,000 people, of whom about one-third were engineers and scientists. Additionally, the Ministry of Agriculture sponsored an extensive R&D program on biological agents against livestock and crop plants; the Ministry of Defense employed thousands of people in several military institutes with expertise in microbiology; the Ministry of Health established a clandestine program to develop biological agents for special operations including assassinations; and the Academy of Sciences of USSR was involved as the highest scientific body in all aspects of the bioweapon programs. The bioweapons program operated under the pretext of producing antibiotics and other biopharmaceutical and veterinary products. As many as 60,000 personnel are estimated to have been involved in various research organizations and production facilities in the Soviet biological weapons programs, all under the watchful surveillance of the KGB. It was typical of those times that scientists and engineers assigned to these programs enjoyed better standards of living than their peers in other areas, but they were prohibited from traveling abroad on business or pleasure (Alibek, 1999; Leitenberg et al., 2012).

The Soviet Union is known to have been engaged in the mass production of numerous pathogens under the aegis of Biopreparat, including and especially *Bacillus anthracis*. In April 1979, an incident occurred at a production facility in Sverdlovsk (today Yekaterinburg) in the southern Ural Mountains that resulted in an outbreak of anthrax causing 68 known deaths. The official explanation from the Soviets was that the outbreak had been caused by contaminated livestock feed, and Soviet authorities refused inspection by Western observers to verify compliance under the auspices of the Biological Weapons Convention. In 1992, after the fall of the Soviet Union, the U.S. government sponsored an investigation of the incident with a team comprised of American and Russian scientists led by Harvard microbiologist Matthew Meselson, which determined the deaths from the Sverdlovsk incident had been caused by the inhalation of anthrax, and not from ingestion as the Soviets had claimed. In fact, the outbreak had been caused by an improperly maintained air filtration system at Compound 19, a military facility operating under Biopreparat, which had accidentally released anthrax spores. Boris Nikolayevich Yeltsin, then President of the Russian Federation, publicly acknowledged that the incident had been caused by an accidental release from a military facility and that the families of the victims would be compensated, though they never were (Alibek, 1999; Guillemin, 1999, 2005; Leitenberg et al., 2012).

Development of multiple antibiotic-resistant strains of *Bacillus anthracis* was alleged to have taken place under the Soviet Biopreparat Program. Other potent, virulent bioweapons developed by the Biopreparat Program included infectious agents such as tularemia (*Francisella tularensis*), brucellosis, typhus (Rickettsia) glanders, melioidosis, Ebola, Marburg, monkey pox, Lassa, Bolivian hemorrhagic fever, Venezuelan equine encephalitis, and smallpox (Variola major).^4^ President Yeltsin avowed in 1992 that the Soviet biological weapons program had been officially terminated, including also the testing facility on Vozrozhdeniya Island in the Aral Sea. Yet, the Russian Federation still possesses repositories of pathogenic bacteria and viruses that were used in the secret biological weapons program, such as the State Scientific Center of Applied Microbiology in Obolensk in the Moscow region, which was reported to have about 3,000 strains of pathogens in its collection. This bioweapon research laboratory complex received western financial aid in the early 2000s for conversion to a peaceful medical manufacturing facility. In the past decade, however, concerns have been voiced by various intelligence agencies regarding the apparent expansion and construction of new laboratory facilities at the site, as well as overtures from Iran to collaborate in the type of research conducted there.

Although the biological warfare arms race was largely a bipolar affair between the U.S. and the Soviet Union during the Cold War years, the People’s Republic of China developed its scientific infrastructure for biotechnology research at this time, allegedly with an eye toward defense against biological weapons. This plan was prioritized, no doubt, with memories fresh in mind of the past attacks and experiments with pathogenic bacteria on Chinese civilians by the notorious Japanese Unit 731 during WWII. The approach in planning, setting up, and equipping research facilities was based on “dual use” of the infrastructure, including defense against bioweapons and capability for bioweapons production for deterrence. China acceded to the Geneva Protocol in 1952. The Chinese government has denied the existence of any active biological warfare program since 1984 when it renounced biological weapons and acceded to the BWC/BTWC. In modernizing facilities, scientific research, and funding in medical, public health, microbiological, and biotechnological sciences, the Chinese government has promoted vaccine programs and emphasized the importance of hygiene for preventing epidemic outbreaks and disease transmission (Schillinger, 2023). The status of research and stockpiles of biological weapons in China is not transparent; however, several reports submitted by China and U.S.-based institutions, agencies, and research centers are readily available on the internet concerning China’s policies and bioweapons R&D, but their factuality may be questionable given the lack of access and inability to authenticate the sources (Smithson, 2007; Croddy, 2022; Mauroni, 2022; U.S. Department of Defense, 2023; Crowley and Dando, 2024).

## The post-Cold War years: the Era of asymmetry, bioterror, and environmental remediation

The fall of the Berlin Wall in November 1989 signaled the imminent demise of the Eastern Bloc, and the collapse of the Soviet Union in December 1991 effectively ended the Cold War, at least between the U.S. and the USSR. The end of the bipolar world configuration left the U.S. as the lone superpower at the time, but it would not be long before it had to recalibrate and adjust to a new, multi-polar world in which asymmetrical warfare and terrorism would present unprecedented challenges. In many ways, the old bipolar paradigm was more stable and predictable, particularly where controlling weapons of mass destruction (WMD)—including nuclear, biological, and chemical (NBC)—were concerned. The signing of the Nuclear Non-Proliferation Treaty in 1968, the BWC in 1972, and the conclusion of the Chemical Weapons Convention (CWC) in 1993, all indicated a collective international will to restrict, if not outright ban, NBC weapons. The CWC regime appeared to offer a model *modus vivendi* for managing international control of WMD with mandatory inspections, verification, and an enforcement agency, which were all components that the Geneva Protocol and BTWC lacked. Such institutional safeguards did not exist for biological weapons, despite the BTWC and the U.S. and many other nations finally ratifying the Geneva Protocol of 1925. There was simply no way to predict or control what rogue nations, terrorist groups, or individuals might do (Guillemin, 2005).

Iraq’s invasion of Kuwait in August 1990 raised new concerns about “niche nations” and their possession of WMDs. During the Iran-Iraq War (1980–1988), under the leadership of Saddam Hussein, Iraq employed chemical weapons against enemy forces, including mustard gas, sarin, and possibly VX (a thiophosphonate enantiomer) with horrific results, and infamously even used them against its own citizens—ethnic Kurds—in a chemical weapons attack on Halabja in 1988. Consequently, as the U.S. led a coalition of United Nations forces to repel the Iraqi invasion of Kuwait in Operation Desert Storm in January 1991, a major concern was whether Saddam Hussein would resort to using chemical or biological weapons. British and U.S. forces were immunized against anthrax, which was thought to be the most likely biological weapon to be used by the Iraqi military. Following the defeat of Iraq in the first Gulf War, in April 1991, the U.N. passed Security Council Resolution 687 (United Nations, 2004), which required Iraq to submit to a regime of international inspections to remove or destroy existing stockpiles of chemical and biological weapons and its capacity to produce them.

While Iraq agreed to inspection by the UNSCOM, it terminated the biological weapons program and declared all biological warfare agents had been destroyed before the UNSCOM could verify the full extent of Iraq’s capabilities. Among the biological agents ostensibly destroyed were *Bacillus anthracis*, *Clostridium botulinum*, *Clostridium perfringens*, aflatoxins, and ricin. Some of these agents had been weaponized in aerial bombs, aerosol sprays, and missile warheads, but UNSCOM could not fully confirm their numbers and destruction. Subsequent UNSCOM efforts to verify clandestine Iraqi stockpiles and negotiate their destruction from late 1991 through 1995 were largely unsuccessful and effectively hampered by the Iraqi government on various pretenses. But UNSCOM was slowly collecting evidence to implicate Iraq and, in July 1995, its government was finally compelled to reveal that it had indeed developed a biological warfare program for offensive purposes, the origins of which dated back to as early as 1974. From these early explorations into biological agents, Iraq developed a significant biological weapons program under the leadership of microbiologist Nassir al Hindawi (Ph.D. 1969, Mississippi State University in Starkville), ultimately leading to the construction of a bioweapons mass production site at Al-Hakam. Effective from the year 2000, the United Nations Monitoring, Verification and Inspection Commission (UNMOVIC) replaced UNSCOM and was mandated by the UN Security Council to monitor the elimination of weapons of mass destruction in Iraq (Guillemin, 2005; Pearson, 2006; Spiers, 2010; Trevan, 2016).

In addition to unpredictable state actors, there are also “rogue nations” and terrorist groups. Beyond Iraq, many other nations in the Middle East and other developing regions—not coincidentally often also being politically unstable parts of the world—have explored development of biological weapons to maintain a strategic balance vis-à-vis other states, including Bulgaria, China, Cuba, Egypt, India, Iran, Israel, Laos, Libya, North Korea, South Africa, South Korea, Syria, Taiwan, and Vietnam, among others (Hunger et al., 2013). The incentives of these countries to develop biological weapons have ranged from deterrence to compensation for weak conventional forces to counter stronger opponents, and intimidation for political and regional hegemony. In addition to bombs and other explosives, international terrorist organizations such as ISIS (Islamic State of Iraq and Syria) and the al-Qaeda network, have threatened and used chemical and biological weapons to destabilize governments for radical political and religious purposes, underscoring the need to establish international barriers for prevention and protection against terrorism (Robinson, 1993; Juergensmeyer, 2003; Salama and Hansell, 2005; Zubay, 2005; Tucker, 2006, 2013).

In the aftermath of the September 11, 2001, terrorist attacks, the potential of chemical and biological terrorism became a timely political and legislative issue. This issue was exacerbated by an apparent biological weapons terrorist attack using anthrax, which occurred in the wake of the 9/11 attacks in early October 2001. In the ensuing weeks, several other cases surfaced as the result of the deliberate release of anthrax (Guillemin, 2011). As a result, on November 25, 2002, the Department of Homeland Security (2023) was established and several bills dealing with measures and protection against terrorism were introduced to the House of Representatives and the Senate. The Science and Technology Directory of the Homeland Department is now charged with defensive programs against biological and chemical threats. The Biowatch Program, started in 2003 to detect and respond to bioterrorism, is an initiative that collaborates and coordinates with networks of public health, emergency management, law enforcement, laboratory, scientific, and environmental health organizations in the U.S. Among other priority areas are intelligence and surveillance and rapid preparedness in the Department of Defense for medical countermeasures (immunization, medical tests, drugs) to eliminate threats in biological warfare especially (Biowatch, 2003, 2011).

These are in addition to the Office of Chemical and Biological Weapons Affairs (OCBWA), established in 2010 as part of the reorganization of the State Department’s arms control agencies, and now charged with the mission to develop policies “to address emerging chemical weapons issues and challenges, assess compliance with the CWC and the Biological Weapons Convention (BWC),” and which now “serves as the U.S. National Authority overseeing U.S. implementation of the CWC” (U.S. Department of State, 2022). Another important measure taken at this time was the implementation of the United Nations Secretary-General’s Mechanism (UNSGM) as a legal instrument for member nations to initiate an investigation of alleged uses of chemical and biological warfare (United Nations, 1987). While this is not maintained as a standing body, member nations may activate and deploy experts of their choosing to conduct the investigation on an *ad hoc* basis.^5^ In the wake of the 9/11 terrorist attacks, in 2004 the UN Security Council passed “Resolution 1540,” which stipulates that “all States shall refrain from providing any form of support to non-State actors that attempt to develop, acquire, manufacture, possess, transport, transfer or use nuclear, chemical or biological weapons and their means of delivery” and “shall take and enforce effective measures to establish domestic controls to prevent the proliferation of weapons and their means of delivery,” a measure taken largely to prevent terrorists from acquiring weapons of mass destruction (United Nations Resolution 1540).^6^ Another example of federal incentives to advance knowledge for public welfare in this area, the CDC has launched a national program to train scientists in emerging infectious diseases that relate to issues of bioterrorism.^7^ On a global scale, the WHO has instituted similar international programs for scientists from developing nations (openwho.org/channels/cbde).

Beyond concerns over rogue nations and terrorist groups, there is always the potential for a natural outbreak of an epidemic or pandemic, for which all nations must remain on guard. The outbreak of the SARS-CoV-2 (severe acute respiratory syndrome coronavirus 2) pandemic in 2019 is a case in point (Himmel and Frey, 2022). Whether the outbreak was naturally occurring and zoonotic in origin (in this case the mutation of the virus in a bat—its original host—to a pangolin) and spread from the Huanan Seafood Wholesale Market, more widely known as the “wet market” in Wuhan, or whether the virus escaped or was deliberately released from the Wuhan Institute of Virology (WIV), which is less than ten miles (14 km) from the Huanan market, remains uncertain and a point of contention. But some facts are not in dispute. The SARS-CoV-2 virus was originally isolated in the Wuhan Institute of Virology and was subsequently isolated and characterized in other countries (Nie, 2020; Stelzer-Braid et al., 2020; Wu et al., 2020; Himmel and Frey, 2022). The WIV is one of twenty separate biomedical research institutes in the Chinese Academy of Sciences, but it is the only institute specializing in virology, viral pathology, and virus technology. The Institute is comprised of five research centers, including the Center for Emerging Infectious Diseases, the Chinese Virus Resources and Bioinformatics Center, the Center of Applied and Environmental Microbiology, the Department of Molecular Virology, and the Department of Analytical Biochemistry and Biotechnology. Although the WIV is ostensibly independent of the People’s Liberation Army (PLA), the PLA retains authority over all bioweapons R&D, related intelligence work, and biological weapons policy in China, and PLA scientists are known to have conducted research in virology and vaccine-related work at the WIV, including vaccines and therapeutics relevant to coronaviruses. Documentation and assessments of China’s life sciences and biotechnology are readily available in various reports (e.g., Shoham, 2015; ODNI, 2023; Rolland, 2024).^8^

An investigation conducted in 2021 by the World Health Organization into the potential role of the Wuhan Institute in the pandemic did not find an unambiguous natural source for the COVID-19 virus, but it also did not uncover evidence for its release from the laboratory. To date, Chinese authorities have turned down requests from the WHO to share information on the animals sold at Wuhan markets, R&D and biosafety conditions at WIV laboratories, and genetic sequences from patients with COVID-19 early in the pandemic (World Health Organization [WHO], 2025). In 2023, the U.S. Office of National Intelligence declassified an intelligence report titled “Potential Links between the Wuhan Institute of Virology and the Origin of the COVID-19 Pandemic,” which did not provide any conclusive insight into distinguishing between a zoonotic source occurring naturally and a laboratory origin of the virus. More recently, the U.S. Central Intelligence Agency has leaned in the direction of favoring the “lab leak” hypothesis, though this conclusion was tagged with a “low confidence” evaluation by its own analysts (Klepper, 2025). Adding fuel to the fire of speculation are documented cases of previous outbreaks. Two epidemic outbreaks of hemorrhagic viruses in the late 1980s in China prompted worldwide speculation concerning the accidental release of Marburg or Ebola-type agents from the Wuhan facility. The outbreak of the virus causing the original form of Severe Acute Respiratory Syndrome (SARS), which was first detected in November 2002 and spread to 28 different countries ranging from Asia to Europe and North America, resulted in about 8,500 infections and nearly 800 fatalities, which only added to concerns about China’s public health policies and its activities in the biological weapons field (Biao and Wong, 2003; Knobler et al., 2004).

In response to the 2019 pandemic, nations around the world have been forced to reexamine their public policy and public health response plans and infrastructure to meet such future challenges. For example, in 2023, the government of the U.K. revised and updated its “UK Biological Security Strategy” policy statement to reflect the realities of a post-COVID-19 world. With new visions of the mission, outcomes and plans, the policy paper outlines five critical areas of biological threats and risks: “a major health crisis (such as pandemic influenza or new infectious disease); antimicrobial resistance; a deliberate biological attack by state or non-state actors (including terrorists); animal and plant diseases, which themselves can pose risks to human health; and accidental release and dual-use research of concern.” The policy paper also outlines the development of a strategic network with multiple outcomes that will help make the U.K. more resilient to a broad spectrum of health risks and threats by 2030 (Gov. U.K., 2023).

Another issue of recent concern is the environmental remediation of former biological weapons R&D facilities and test sites. On Gruinard Island, the site of the U.K.’s extensive anthrax testing in 1942 and 1943, a massive decontamination effort was undertaken from 1979 using 280 tons of formaldehyde as a sterilant and 2,000 tons of seawater to clean up hot spots on the island. Other sporicidal chemicals were also tested for soil decontamination: potassium permanganate, glutaraldehyde, peracetic acid, and dodecylamine (Manchee et al., 1983). The decontamination program was finalized with an intentional fire that burned the brush and other vegetation. While *Bacillus anthracis* spores are reputedly still found in samples from Gruinard Island, they are not perceived to constitute a public health hazard because they are embedded under the topsoil. In 1990, the Ministry of Defence declared Gruinard Island anthrax free, and ownership of the island was returned in 1990 to the heirs of the original owner, who subsequently sold it to another private landowner in Scotland (Willis, 2009).

The former island of Vozrozhdeniya (now a peninsula) in the Aral Sea was one of the Soviet Union’s main open-air biological testing sites and, today, is part of the independent Republic of Uzbekistan. The legacy of testing at Vozrozhdeniya is an environmental catastrophe with hundreds of tons of anthrax bacterial biomass disposed of by burial in sediment layers. With the water level receding in the Aral Sea, some burial sites have come very close to surfacing, causing concern about potential leakage of the storage drums and transmission to wildlife. As part of the economic package and cooperation stemming from the September 11, 2001, terrorist act in New York City, and the subsequent international effort to curtail terrorism, the U.S. offered to help the Uzbekistan government to clean up and dispose of the anthrax containers in a safe manner. Funding was also provided for travel to the U.S. and training in a broad range of programs covering education, energy, environment, and democracy, among many other issues.^9^

## Conclusion: the mixed legacy of the Geneva Protocol

There seems to be no general consensus among historians or policymakers on the efficacy of the Geneva Protocol of 1925. Even as the Protocol was being drafted and the process of ratification was underway, observers expressed serious concerns about the prospects of its success without the full commitment of all parties (Hudson, 1924). In 1937, U.S. Brigadier General Agustin M. Prentiss, Technical Director of the Edgewood Arsenal and a member of the Chemical Warfare Service (CWS) in World War I, noted the lack of enthusiasm the international community seemed to have for the agreement, quipping “its apathetic reception by various governments has tended to defeat the purpose it was expected to serve” (Prentiss, 1937; Croddy, 2005). Ironically, perhaps no organization was more responsible for undermining the potential force of the Protocol than the U.S. Chemical Warfare Service itself, particularly under the leadership of General Amos A. Fries, who served as director of the CWS from 1919 to 1929 and effectively mobilized a combined lobby of chemists of the American Chemical Society and former members of the CWS to kill ratification of the Protocol in the U.S. Senate over fears of the impact it would have on the American chemical industry and diminishing the influence of the CWS. Moreover, they feared the League of Nations would eventually be empowered to influence control over the manufacture of chemicals if the Protocol were ratified, and the relationship between President Calvin Coolidge’s administration (1923–1929) and the League of Nations was arguably “ambivalent” at best (Jones, 1980; McElroy, 1991). Without Senate ratification, the U.S. professed support for the Protocol rang hollow and undermined its potential.

Aside from a reluctance to become further entangled with the League of Nations, the impetus for which came largely from an influential cadre of U.S. senators collectively known as “bitter-enders” or “irreconcilables” because of their irreconcilable opposition to participation in the League, Coolidge was also ambivalent about the Protocol because it “did not include any monitoring mechanisms or enforcement structures, relying instead on the good behavior of individual nations,” rendering it little more than a “gentlemen’s agreement” among states as it were, a systemic weakness inherent in the League structure itself (McElroy, 1991). Furthermore, U.S. Secretary of State Frank B. Kellogg expressed the government’s reluctance to submit to the League’s authority on the matter, stating, “The United States will not tolerate the supervision of any outside body in this matter nor be subject to inspection or supervision by foreign agencies or individuals.” In the end, the failure of the Coolidge administration to ratify the Protocol ultimately rested with the president himself (McElroy, 1991).

Because the Protocol did not prohibit the research, development, production, or stockpiling of chemical or biological weapons, it was effectively reduced to a “no-first-use” prohibition. But as the brief history presented above has shown, even this prohibition failed to stop chemical and biological weapons from being used from the 1930s and beyond. The Protocol failed to prevent Italy’s use of mustard gas against Abyssinian forces in 1935, Japan’s widespread use of chemical and biological weapons throughout China and its Asian empire, U.S. use of defoliants (Agent Orange) and irritant chemicals (tear gas) in Vietnam, Egypt’s use of nerve gases in Yemen in 1967, or Iraq’s use of chemical weapons against Iran and its own ethnic Kurd population in the 1980s, all of which were arguably flagrant violations of the Geneva Protocol of 1925. Nor has the Protocol prevented the use of poison gases or toxins by terrorists or assassins, as in the 1995 sarin gas attack on the Tokyo subway system launched by members of the Aum Shinrikyo Cult, the attempted assassination of U.S. congress members with anthrax in 2001, or the assassination of Alexander Litvinenko, a former KGB member and defector who was poisoned with a lethal dose of radioactive ^210^Po in 2006 (Tucker, 2006; Dyer, 2007).

History suggests that what mattered most in the context of war was not the legal prohibition of CBW as defined in the 1925 Geneva Protocol, but *deterrence*, that is, the ability of the defender to retaliate in kind. In most cases described above, the aggressor nations engaged in chemical and biological warfare without concern that these attacks would be returned in kind. The Protocol itself was powerless to prevent these attacks and served only as a legal constraint for ratifying nations who may or may not be held to account depending upon the outcome of the given war. Like the League of Nations itself, the Geneva Protocol had “no teeth,” no real powers of monitoring, verification, or enforcement. Consequently, new protocols were required to address these shortcomings, which ultimately led to the Biological and Toxin Weapons Convention of 1975 and the Chemical Weapons Convention of 1993. As chemical and biological warfare scholar Eric A. Croddy wrote in 2005, “With the 1993 Chemical Weapons Convention now in force, the Geneva Protocol is mostly only relevant today in its prohibition of biological warfare…,” but “glaring loopholes” remain, and the BTWC itself has remained in a state of relative limbo, “awaiting some initiative to achieve consensus among its parties” (Croddy, 2005).

On the other hand, others argue that the Geneva Protocol and the BTWC should not be evaluated in isolation as they generally serve as the “core elements” of a wider anti-biological weapons regime consisting of “a range of agreements and mechanisms implemented by States, [and] non-State actors in industry and civil society.” Policy analyst Jez Littlewood illustrates how the BTWC has evolved incrementally through regular “review conferences” of concerned states parties that have convened every 5 years since 1980 resulting in over 140 additional “understandings” regarding the agreement and how to implement it. While the Geneva Protocol and BTWC serve as “the legal basis for a complete prohibition on the development, production, stockpiling, acquisition and use of biological and toxin weapons,” the additional understandings, while not legally binding, “represent a road map to the what and how of biological disarmament” (Littlewood, 2024).

As of August 2025, 189 states are now signatories of the BTWC, and “the norm against biological weapons use has become nearly universally accepted” (Wikipedia BTWC, Cross and Klotz, 2020). The convention largely strives to compensate for prohibitions not entailed in the original Protocol, that is, “the convention bans only the development, production, and stockpiling of biological agents (including toxins) for purposes and in quantities that have no justification for peaceful purposes as well as the development and possession of weapons systems for dissemination of biological agents,” but “it does not outlaw the wartime use of biological weapons; that’s banned in the Geneva Protocol of 1925” (Cross and Klotz, 2020, UNODA). Unlike chemical weapons programs, which under the CWC are now subject to verification and inspection regimes, it is much more problematic to apply these to biological weapons programs given the particular “dual use” characteristics of bacteria and viruses for medical and life science research. It also does not serve as a deterrent to terrorists or criminals as “biocrimes”—the use of biological agents in acts of terrorism or assassination—which remain outside the BTWC mandate as well (Thränert, 1996; Cross and Klotz, 2020).

Recent evaluations of the BTWC may be as mixed as those of the Geneva Protocol, with some critics emphasizing its inability to prevent the development of biological agents and their use by terrorists, while others insist that having established a “nearly universal norm against biological weapons” and with the diplomatic community having largely addressed remaining concerns, the BTWC is now “far from being a toothless paper tiger” (Beard, 2007; Cross and Klotz, 2020). As it stands today, however, the most readily apparent legacy of the 1925 Geneva Protocol would seem to be its ban on the use of chemical and biological agents and toxins in *warfare*, a prohibition by which most—*but not all*—nations have abided. But there seems to be no clear consensus on the extent to which this prohibition has been internalized by the international community (Evangelista and Tannenwald, 2017). It took another major agreement, the BTWC, and the subsequent “understandings,” to bring the biological weapons control regime more in line with the CWC and nuclear non-proliferation and disarmament treaties. On the whole, however, the prohibition against biological weapons still remains largely “a gentlemen’s agreement,” a problem that the BTWC also could not solve. In the long run, the extent to which the Geneva Protocol succeeded or failed in the objective to prevent chemical and biological warfare remains a point of debate among historians and policymakers even 100 years after its presentation to the League of Nations in 1925.

## Footnotes

1
www.bbc.com/news/world-asia-china-68705857

2
https://www.gov.uk/government/news/foot-and-mouth-disease-latest-situation

3
https://history.state.gov/milestones/1861-1865/blockade

4
https://thebulletin.org/2022/10/the-russian-biological-weapons-program-in-2022/

5
https://web.archive.org/web/20220312135907/https://www.un.org/disarmament/wmd/secretary-general-mechanism/

6
https://digitallibrary.un.org/record/520326?v=pdf

7
https://www.cdc.gov/orr/index.html

8
www.dni.gov/index.php/120-dni

9
https://2009-2017.state.gov/p/eur/rls/rpt/23630.htm

## References

[B1] Aldhous P. (1990). Biological warfare. Gruinard Island handed back. *Nature* 344:801. 10.1038/344801b0 2109833

[B2] Alibek K. (1999). *Biohazard: The chilling true story of the largest covert biological weapons program in the world – told from the inside by the man who ran it.* New York, NY: Random House.

[B3] Balmer B. (2001). *Britain and biological warfare: Expert advice and science policy, 1930-65.* New York, NY: Palgrave.

[B4] Beard J. M. (2007). The shortcomings of indeterminacy in arms control regimes: The case of the Biological Weapons Convention. *Am. J. Int. Law* 101 271–321. 10.1017/S0002930000030098

[B5] Biao X. Wong T. (2003). SARS: Public health and social science perspectives. *Econ. Polit. Weekly* 38 2480–2483.

[B6] Biowatch (2003). “The Biowatch Program: Detection of bioterrorism,” in *Proceedings of the Congressional Research Service Report RL32152*, (South Africa: Biowatch).

[B7] Biowatch, (2011). “Institute of medicine (US) and national research council (US) committee on effectiveness of national biosurveillance systems: Biowatch and the Public Health System,” in *Biowatch and public health surveillance: Evaluating systems for the early detection of biological threats: Abbreviated version*, (Washington, D.C: National Academies Press). 25032347

[B8] Braterman P. S. (2012). “Science, war, and morality; The tragedy of Fritz Haber,” in *From to stars to stalagmites. How everything connects*, (Singapore: World Scientific Publishing Co).

[B9] Brophy L. P. Miles W. D. Cochrane R. C. (1959). *The chemical warfare service: From laboratory to field. United States Army in World War II: The technical services.* Arlington, VA: Office of the Chief of Military History, Department of the Army.

[B10] Brown F. J. (2006). *Chemical warfare: A study in restraints.* Piscataway, NJ: Transaction Publishers.

[B11] Bush L. M. Perez M. T. (2012). The anthrax attacks 10 years later. *Ann. Intern. Med.* 156 41–44. 10.7326/0003-4819-155-12-201112200-00373 21969275

[B12] Butler D. (1997). US-Cuba row over insects goes to weapons meeting. *Nature* 388:705. 10.1038/41846

[B13] Carter G. Balmer B. (1999). “Chemical and biological warfare and defense, 1945-90,” in *Cold war, hot science: Applied research in Britain’s defence laboratories*, eds Bud R. Gummett P. (Amsterdam: Harwood Academic Publishers), 295–338.

[B14] Carter G. B. (1992). Biological warfare and biological defence in the United Kingdom 1940-1979. *RUSI J.* 137 67–74. 10.1080/03071849208445664

[B15] Chai P. R. Hayes B. D. Erickson T. B. Boyer E. W. (2018). Novichok agents: A historical, current, and toxicological perspective. *Toxicol. Commun.* 2 45–48. 10.1080/24734306.2018.1475151 30003185 PMC6039123

[B16] Cole L. A. (1988). *Clouds of secrecy: The Army’s germ warfare tests over populated areas.* Langham, MD: Rowman and Littlefield.

[B17] Cole L. A. (1997). *The eleventh plague: The politics of biological and chemical warfare.* New York, NY: W. F. Freeman and Co.

[B18] Cookson J. Nottingham J. (1969). *A Survey of Chemical and Biological Warfare.* New York, NY: MR Press.

[B19] Croddy E. (2022). China’s role in the chemical and biological disarmament regimes. *Nonprolif. Rev.* 9 16–47. 10.1080/10736700208436872

[B20] Croddy E. A. Perez-Armendariz C. Hart J. (2002). *Chemical and biological warfare: A comprehensive survey for the concerned citizen.* New York, NY: Copernicus Books.

[B21] Croddy E. A. (2005). “Geneva protocol,” in *Weapons of mass destruction: An encyclopedia of worldwide state policy, technology, and history: Chemical and biological weapons*, eds Croddy E. A. Wirtz J. J. (Santa Barbara, CA: ABC-CLIO), 140–143.

[B22] Cross G. Klotz L. (2020). Twenty-first century perspectives on the biological weapon convention: Continued relevance or toothless paper tiger. *Bull. Atomic Scient.* 76 185–191. 10.1080/00963402.2020.1778365

[B23] Crowley M. Dando M. (2024). Regulation of toxins and bioregulators under the chemical weapons convention and the biological and toxin weapons convention. *J. Biosaf. Biosecur.* 6 99–112. 10.1016/j.jobb.2024.03.003

[B24] Department of Homeland Security (2023). *Biological incident annex to the response and recovery. Federal interagency operational plan.* Washington, D.C: Department of Homeland Security.

[B111] Dorsey M. G. (2024). *Holding their breath: How the Allies confronted the threat of chemical warfare in World War II*. Ithaca, NY: Cornell University Press.

[B25] DSTL (2025). *Defence Science and Technology Laboratory, Annual Report and Accounts 2024-2025.* Salisbury: DATL.

[B26] Dyer O. (2007). More cases of polonium-210 contamination are uncovered in London. *Brit. Med. J.* 334:65. 10.1136/bmj.39091.604641.DB 17218691 PMC1767288

[B27] Evangelista M. Tannenwald N. (2017). *Do the Geneva Conventions Matter?* Oxford: Oxford University Press.

[B28] Geissler E. van Courtland Moon J. E. (1999). *Biological and toxin weapons: Research, development and use from the middle ages to 1945.* Oxford: Oxford University Press.

[B29] Gov. U.K. (2023). *UK biological security strategy: Policy paper.* Available online at: www.gov.uk/government/publications/uk-biological-security-strategy (accessed September 17, 2025).

[B30] Grunden W. E. (2005). *Secret weapons and World War II: Japan in the shadow of big science.* Lawrence, KS: University Press of Kansas.

[B31] Guillemin J. (1999). *Anthrax: The investigation of a deadly outbreak.* Oakland, CA: University of California Press.

[B32] Guillemin J. (2005). *Biological weapons: From the invention of state-sponsored programs to contemporary bioterrorism.* New York, NY: Columbia University Press.

[B33] Guillemin J. (2011). *American anthrax: Fear, crime, and the investigation of the nation’s deadliest bioterror attack.* New York, NY: Times Books.

[B34] Guillemin J. (2017). *Hidden atrocities: Japanese germ warfare and American obstruction of justice at the Tokyo trial.* New York, NY: Columbia University Press.

[B35] Hammond P. Carter G. (2002). *From biological warfare to healthcare: Porton Down, 1940-2000.* London: Palgrave.

[B36] Harris R. Paxman J. (2002). *A higher form of killing: The secret history of chemical and biological warfare.* New York, NY: Random House.

[B37] Harris S. (1994). *Factories of death: Japanese biological warfare 1932-45 and the American cover-up.* Milton Park: Routledge.

[B38] Hay A. (2007). A magic sword or a big itch: An historical look at the United States Biological Weapons Programme. *Med. Confl. Surv.* 15 215–234. 10.1080/13623699908409460 10472190

[B39] Hersh S. (1968). *Chemical and biological warfare: America’s hidden arsenal.* Indianapolis, IN: Bobbs-Merrill.

[B40] Himmel M. Frey S. (2022). SARS-CoV-2: International investigation under the WHO or BWC. *Front. Publ. Health* 9:636679. 10.3389/fpubh.2021.636679 35186855 PMC8850392

[B41] Hudson M. O. (1924). The Geneva Protocol. *For. Aff.* 3 226–235.

[B42] Humphreys J. M. Stenfeldt C. King D. P. Knight-Jones T. Perez A. M. VanderWaal K. (2025). Epidemiology and economics of foot-and-mouth disease: Current understanding and knowledge gaps. *Vet. Res.* 56:141. 10.1186/s13567-025-01561-5 40624570 PMC12235859

[B43] Hunger I. Radosavljevic V. Belojevic G. Rotz L. D. (eds) (2013). *Biopreparedness and public health: Exploring synergies.* Dordrecht: Springer.

[B44] Jones D. P. (1980). American chemists and the Geneva Protocol. *ISIS* 71 426–440. 10.1086/352542

[B45] Juergensmeyer M. (2003). *Terror in the mind of god: The global rise of religious violence.* Berkeley, CA: University of California Press.

[B46] Klepper D. (2025). *The CIA believes covid most likely originated from a lab but has low confidence in its own finding.* Available online at: apnews.com/article/covid-cia-trump-china-pandemic-lab-leak-9ab7e84c626fed68ca13c8d2e453dde1 (accessed January 26, 2025).

[B47] Knight-Jones T. J. D. Rushton J. (2013). The economic impacts of foot and mouth disease – What are they, how big are they and where do they occur? *Prevent. Vet. Med.* 112 161–173. 10.1016/j.prevetmed.2013.07.013 23958457 PMC3989032

[B48] Knobler S. Mahmoud A. Lemon S. Mack A. Sivitz L. Oberholtzer K. (eds) (2004). *Forum on microbial threats learning from SARS: Preparing for the next disease outbreak – workshop summary.* Washington, D.C.: National Academies Press. 22553895

[B49] Koenig R. L. (2006). *The fourth horseman. One man’s secret mission to wage the Great War in America.* New York, NY: PublicAffairs.

[B50] Leitenberg M. (1998). New Russian evidence on the Korean War biological warfare allegations: Background and analysis. *Cold War Int. Hist. Project Bull.* 11 185–199.

[B51] Leitenberg M. (2008). “False allegations of U.S. biological weapons use during the Korean war,” in *Terrorism, war, or disease? Unraveling the use of biological weapons*, eds Clunan A. L. Lavoy P. R. Martin S. B. (Stanford, CA: Stanford University Press), 120–143.

[B52] Leitenberg M. Zilinskas R. A. Kuhn J. H. (2012). *The Soviet biological weapons program: A history.* Cambridge, MA: Harvard University Press.

[B53] Littlewood J. (2024). “The 1925 Geneva protocol and the BTWC,” in *Essentials of biological security: A global perspective*, eds Shang L. Zhang W. Dando M. (Hoboken, NJ: John Wiley and Sons), 121–131.

[B54] Lockwood J. A. (2009). *Six-legged soldiers, using insects as weapons of war.* Oxford: Oxford University Press.

[B55] Lockwood J. A. (2012). Insects as weapons of war, terror, and torture. *Annu. Rev. Entomol.* 57 205–227. 10.1146/annurev-ento-120710-100618 21910635

[B56] Lucas G. B. (1980). The war against blue mold. *Science* 210 147–153. 10.1126/science.210.4466 17741271

[B57] Manchee R. J. Broster M. G. Anderson I. S. Henstridge R. M. (1983). Decontamination of *Bacillus anthracis* on Gruinard Island? *Nature* 303 239–240. 10.1038/303239a0 6405284

[B58] Manchee R. J. Broster M. G. Melling J. Henstridge R. M. Stagg A. J. (1981). *Bacillus anthracis* on Gruinard Island. *Nature* 294 254–255. 10.1038/294254a0 6795509

[B59] Materials (1950). *Materials on the trial of former servicemen of the Japanese army charged with manufacturing and employing bacteriological weapons.* Moscow: Foreign Languages Publishing.

[B60] Mauroni A. (2022). On biological war. *Milit. Hist.* 102 28–37.

[B61] McElroy R. J. (1991). “The Geneva protocol of 1925,” in *The politics of arms control treaty ratification*, eds Krepon M. Caldwell D. (New York, NY: St. Martin’s Press), 125–166.

[B62] McFee R. B. Leikin J. B. (2009). Death by polonium-210: Lessons learned from the murder of former Soviet spy Alexander Litvinenko. *Semin. Diagn. Pathol.* 26 61–67. 10.1053/j.semdp.2008.12.003 19292030

[B63] Nehring C. (2021). Active and sharp measures. cooperation between the Soviet KGB and Bulgarian State Security. *J. Cold War Stud.* 23 3–33. 10.1162/jcws_a_01038

[B64] Nie J.-B. (2020). In the shadow of biological warfare: Conspiracy theories on the origins of COVID-19 and enhancing global governance of biosafety as a matter of urgency. *J. Bioeth. Inq.* 17 567–574. 10.1007/s11673-020-10025-8 32840850 PMC7445685

[B65] ODNI (2023). *Office of the Director of National Intelligence. The potential links between the Wuhan Institute of Virology and the origin of the covid-19 pandemic.* Available online at: www.dni.gov/index.php/newsroom/reports-publications/reports-publications-2023/3704-odni-releases-report-on-the-potential-links-between-the-wuhan-institute-of-virology-and-the-origin-of-covid-19 (accessed August 13, 2025).

[B66] O’Flaherty O. (1955). The blockade that failed. *Am. Herit.* 6 38–41.

[B67] Oldstone M. B. A. (2010). *Viruses, plagues, and history: Past, present, and future.* Oxford: Oxford University Press.

[B68] Pearson G. S. (2006). “The Iraqi biological weapons program,” in *Deadly cultures: Biological weapons since 1945*, eds Wheelis M. Rózsa L. Dando M. (Cambridge, MA: Harvard University Press), 169–190.

[B69] Pischel L. Martini B. A. Yu N. Cacesse D. Tracy M. Kharbanda K. (2024). Vaccine effectiveness of 3rd generation mpox vaccines against mpox and disease severity: A systematic review and meta-analysis. *Vaccine* 42:126053. 10.1016/j.vaccine.2024.06.021 38906763

[B70] Prentiss A. M. (1937). *Chemicals in war: A treatise on chemical warfare.* New York, NY: McGraw-Hill.

[B71] Regis E. (1999). *The biology of doom: The history of America’s secret germ warfare project.* New York, NY: Henry Holt and Co.

[B72] Regis E. (2023). “Project 112,” in *Science, secrecy, and the Smithsonian: The strange history of the Pacific Ocean Biological Survey Program*, (Oxford: Oxford University Press), 70–85.

[B73] Robinson J. P. (1993). “Chemical-weapons proliferation in the Middle East,” in *Non-conventional weapons proliferation in the Middle East*, eds Karsh E. Navias M. S. Sabin P. A. G. (Oxford: Clarendon Press), 70–98.

[B74] Robinson J. P. (ed.) (1971). *The rise of CB weapons. Stockholm International Institute for Peace and Conflict Research. The problem of chemical and biological warfare: A study of the historical, technical, military, legal, and political aspects of CBW, and possible disarmament measures.* Atlantic Highland, NJ: Humanities Press.

[B75] Rolland N. (ed.) (2024). *Under the microscope: China’s evolving biotechnology ecosystem. NBR Special Report #114.* Seattle, WA: National Bureau of Asian Research, 52.

[B76] Russell A. Vogler J. (eds) (2000). *The international politics of biotechnology: Investigating global futures.* Manchester: Manchester University Press.

[B77] Salama S. Hansell L. (2005). Does intent equal capability? Al-Qaeda and weapons of mass destruction. *Non-Prolif. Rev.* 12 615–653. 10.1080/10736700600601236

[B78] Salisbury S. Dew K. (2023). Murder on Waterloo Bridge: Placing the assassination of Georgi Markov in past and present context, 1970-2018. *Contemp. Brit Hist.* 37:156. 10.1080/13619462.2022.2160707

[B79] Schillinger N. (2023). Microbic mass destruction: Biological warfare and epidemic prevention in Republican China. *East Asian Sci. Technol. Soc. Int. J.* 17 148–169. 10.1080/18752160.2021.1984009

[B80] Schmaltz F. (2017). “Chemical weapons research on soldiers and concentration camp inmates in Nazi Germany,” in *One hundred years of chemical warfare: Research, deployment, consequences*, eds Friedrich B. Hoffmann D. Renn J. Schmaltz F. Wolf M. (Cham: Springer Nature), 229–257.

[B81] Schmidt U. (2015). *Secret science: A century of poison warfare and human experiments.* Oxford: Oxford University Press.

[B82] Scholthof K.-B. G. Adkins S. Czosnek H. Palukaitis P. Jacquot E. Hohn T. (2011). Top 10 plant viruses in molecular plant pathology. *Mol. Plant Pathol.* 12 938–954. 10.1111/J.1364-3703.2011.00752.X 22017770 PMC6640423

[B83] Shoham D. (2015). China’s biological warfare programme: An integrative study with special reference to biological weapons capabilities. *J. Defen. Stud.* 9 131–156.

[B84] Smithson A. E. (ed.) (2007). *Beijing on biohazards. Chinese experts on bioweapons nonproliferation issues.* Monterey, CA: Monterey Institute of International Studies, 141.

[B85] Spencer R. C. Wilcox M. H. (1993). Agents of biological warfare. *Rev. Res. Med. Microbiol.* 4 138–143.

[B86] Spiers E. M. (1986). *Chemical warfare.* London: Palgrave MacmiIllan.

[B87] Spiers E. M. (2010). *A history of chemical and biological weapons.* London: Reaktion Books.

[B88] Stelzer-Braid S. Walker G. J. Aggarwal A. Isaacs S. R. Yeang M. Naing Z. (2020). Virus isolation of severe acute respiratory syndrome coronavirus 2 (SARS-CoV-2) for diagnostic and research purposes. *Pathology* 52 760–763. 10.1016/j.pathol.2020.09.012 33131800 PMC7543926

[B89] Thränert O. (ed.) (1996). *Enhancing the biological weapons convention.* Bonn: Verlag J.H.W.

[B90] Trevan T. (2016). “The Iraqi biological warfare program,” in *Biological threats in the 21st Century*, ed. Lentzos F. (London: Imperial College Press), 113–129.

[B91] Tucker J. B. (2001). *Scourge: the once and future threat of smallpox.* New York, NY: Grove Press.

[B92] Tucker J. B. (2006). *War of nerves: Chemical warfare from world war I to Al-Qaeda.* New York, NY: Anchor Books.

[B93] Tucker J. B. (2013). “The current bioweapons threat,” in *Biopreparedness and public health: Exploring synergies*, eds Hunger L. Radosavljevic V. Belojevic G. Rotz L. D. (Dordrecht: Springer), 7–16.

[B94] U.S. Department of Defense (2023). *Biodefence posture review.* Arlington County, VA: U.S. Department of Defense.

[B95] U.S. Department of State (2022). *Office of Chemical and Biological Weapons Affairs. Mission statement.* Washington, D.C: U.S. Department of State.

[B96] U.S. Department of the Army (1977). *Army activity in the U.S.: Biological warfare programs. Department of the Army report, with appendices.* Arlington County, VA: U.S. Department of the Army.

[B97] United Nations (1987). *Office for Disarmament Affairs. (UNODA) Secretary-General’s Mechanism for investigation of alleged use of chemical and biological weapons (UNSGM).* New York, NY: United Nations.

[B98] United Nations (2004). *Security Council. (UNSC) Resolution 1540. Adopted 28 April 2004 at the 4956*^th^* Meeting of the UNSC.* New York, NY: United Nations.

[B99] van Courtland Moon E. J. (2009). “The US biological weapons program,” in *Deadly cultures: Biological weapons since 1945*, eds Wheelis M. Rózsa L. (Cambridge, MA: Harvard University Press), 9–46.

[B100] Vázquez L. L. Rodriguez E. (1999). Plantas hospedantes de *Trips palmi* Karny (Thysanoptera: Thripidae) en Cuba. *Fitosanidad* 3 37–40.

[B101] Wheelis M. Rózsa L. Dando M. (eds) (2006). *Deadly cultures: Biological weapons since 1945.* Cambridge, MA: Harvard University Press.

[B102] Whitby S. M. (2002). “Anti-crop BW and the BTWC,” in *Biological warfare against crops*, (London: Palgrave Macmillan), 43–65.

[B103] Williams P. Wallace D. (1989). *Unit 731: Japan’s secret biological warfare in World War II.* New York, NY: Free Press.

[B104] Willis E. A. (2009). Landscape with dead sheep: What they did to Gruinard Island. *Med. Confl. Surv.* 25 320–331. 10.1080/13623690903417382 20178200

[B105] Willstätter R. (1965). “In memory of Fritz Haber 1965,” in *From my life. The memoirs of Richard Willstätter*, (New York, NY: W.A. Benjamin), 255–293.

[B106] World Health Organization [WHO] (2025). *Independent assessment of the origins of SARS-CoV-2. Scientific Advisory Group for the origins of novel pathogens (SAGO).* Geneva: WHO.

[B107] Wu D. Wu T. Liu Q. Yang Z. (2020). The SARS-CoV-2 outbreak: What we know. *Int. J. Infect. Dis.* 94 44–48. 10.1016/j.ijid.2020.03.004 32171952 PMC7102543

[B108] Zilinskas R. A. (1999). Cuban allegations of biological warfare by the United States: Assessing the evidence. *Crit. Rev. Microbiol.* 25 173–227. 10.1080/10408419991299202 10524329

[B109] Zilinskas R. A. (ed.) (2000). *Biological warfare: Modern offense and defense.* Boulder, CO: Lynne Rienner Publishers.

[B110] Zubay G. (ed.) (2005). *Agents of bioterrorism: Pathogens and their weaponization.* New York, NY: Columbia University Press.

